# Rapid detection of copy number variations and point mutations in *BRCA1/2* genes using a single workflow by ion semiconductor sequencing pipeline

**DOI:** 10.18632/oncotarget.26000

**Published:** 2018-09-14

**Authors:** Aldo Germani, Fabio Libi, Stefano Maggi, Gianluca Stanzani, Augusto Lombardi, Patrizia Pellegrini, Mauro Mattei, Laura De Marchis, Claudio Amanti, Antonio Pizzuti, Maria Rosaria Torrisi, Maria Piane

**Affiliations:** ^1^ Department of Clinical and Molecular Medicine, “Sapienza” University of Rome, Rome, Italy; ^2^ Sant’Andrea University Hospital, Rome, Italy; ^3^ Department of Medical and Surgical Sciences and Translational Medicine, Rome, Italy; ^4^ Department of Radiation, Anatomopathological, Oncological Science, “Sapienza” University of Rome, Rome, Italy; ^5^ Department of Experimental Medicine, “Sapienza” University of Rome, Rome, Italy; ^6^ Clinical Genomics Unit, IRCCS Casa Sollievo della Sofferenza, San Giovanni Rotondo, Italy

**Keywords:** CNV, NGS, MLPA, MAQ, BRCA genes

## Abstract

Molecular analysis of *BRCA1* (*MIM*# *604370)* and *BRCA2* (*MIM* #600185) genes is essential for familial breast and ovarian cancer prevention and treatment. An efficient, rapid, cost-effective accurate strategy for the detection of pathogenic variants is crucial. Mutations detection of *BRCA1/2* genes includes screening for single nucleotide variants (SNVs), small insertions or deletions (indels), and Copy Number Variations (CNVs). Sanger sequencing is unable to identify CNVs and therefore Multiplex Ligation Probe amplification (MLPA) or Multiplex Amplicon Quantification (MAQ) is used to complete the *BRCA1/2* genes analysis. The rapid evolution of Next Generation Sequencing (NGS) technologies allows the search for point mutations and CNVs with a single platform and workflow. In this study we test the possibilities of NGS technology to simultaneously detect point mutations and CNVs in *BRCA1/2* genes, using the Oncomine^TM^ BRCA Research Assay on Personal Genome Machine (PGM) Platform with Ion Reporter Software for sequencing data analysis (Thermo Fisher Scientific). Comparison between the NGS-CNVs, MLPA and MAQ results shows how the NGS approach is the most complete and fast method for the simultaneous detection of all *BRCA* mutations, avoiding the usual time consuming multistep approach in the routine diagnostic testing of hereditary breast and ovarian cancers.

## INTRODUCTION

Most cases of breast and ovarian cancers are sporadic. However, up to 20% of patients are likely to harbor a dominant susceptibility allele, with highly increased risk of malignancy above the general population. Many genes, when mutated, increase risk of breast cancer development [1]. Some of them work either as cell cycle controllers in response to DNA damage or in DNA Double-Strand Breaks Repair by homologous recombination; however, the most part of them have low or moderate penetrance, and are rarely involved. Notable exceptions are the *BRCA1* and the *BRCA2* genes, whose mutations have high penetrance and are found in nearly 40% of cancer patients [2–4]. Cumulative cancer risks at age 70 years were breast cancer risk of 57% for *BRCA1* and 49% for *BRCA2* mutation carriers; and ovarian cancer risk of 40% for *BRCA1* and 18% for *BRCA2* mutation carriers. [5, 6]. *BRCA1* and *BRCA2* pathogenic variants include point mutation, small insertion/deletion and large genomic rearrangements (LGRs). Copy Number Variation (CNV) is a structural variation where a DNA segment of 1Kb to several Mb in length is present in variable copies compared to a reference genome sequence [7]. Although these structural variants do not always display phenotypic consequences, sometimes they may influence gene expression and be associated with specific phenotypes [8]. LGRs are responsible for 4-28% of all inherited *BRCA* mutations [9–11]. Pathogenic CNVs in *BRCA1* and *BRCA2* genes range respectively from 0 to 27% and 0% to 8% [12–14] The higher frequency of LGRs in *BRCA1* compared to *BRCA2* gene is caused by higher density of *ALU* sequences [15], which mediate the formation of LGRs [16, 17]. LGRs in *BRCA1* and *BRCA2* vary across populations and ethnicities [18–20], with a frequency up to 12% in Italy [21]. Many approaches have been used for detecting *BRCA* LGRs, such as comparative genomic hybridization (CGH), array comparative genomic hybridization (aCGH), real Time PCR (qPCR) and fluorescent in situ Hybridization (FISH) [22]. In 2006, a test for identifying large rearrangements in these genes (BRAC-Analysis Rearrangement Testing) was released from Myriad. Nowadays, the Multiplex ligation-dependent probe amplification (MLPA, MRC-Holland, Amsterdam, the Netherlands) is the primary method for detecting LGRs in *BRCA1/2* with the Multiplex Amplicon Quantification (MAQ) (Multiplicon, Niel, Belgium), as an alternative way [23–25]. Both approaches are consistent, but they always need the confirmation with other techniques, because of the possibility of false positive results [21]. For many years, *BRCA* gene analysis on peripheral blood has been using Sanger sequencing and MLPA. The Next Generation Sequencing (NGS) had definitively replaced the Sanger method since it is extremely fast and cost-effective as a routine analysis. The rapid evolution of NGS allows to add LGRs to point mutation screening in a single workflow, further reducing the time for the complete analysis of *BRCA1/2* genes. However, NGS-CNV detection has not been validated for clinical diagnostics yet [26, 27]. In this work we evaluated the use of the PGM Ion Torrent platform for the simultaneous identification of CNVs, single nucleotide variants (SNVs) and indels (single or multiple insertion/deletion), using a single integrated workflow. Data obtained from the NGS-CNV detection have been compared with those achieved by MLPA and MAQ. The results show how the NGS workflow on the Ion PGM platform can be used for the identification of any type of pathogenic variants of the *BRCA1/2* genes. This analysis pipeline could be used in diagnostic and predictive tests for hereditary breast and ovary cancer, as this approach proved to be fast, reliable and accurate both in identifying SNVs and indels, and in the simultaneous analysis of CNVs.

## RESULTS

### Mutational data

**Figure 1:**
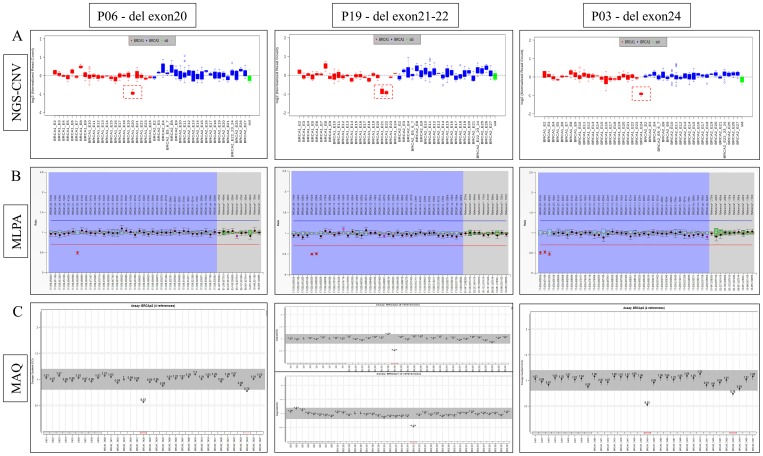
Large rearrangements identified in P06, P19, P03 patients by NGS-CNV workflow respectively in exon 20, 21-22 and 24 of *BRCA1* gene compared with MLPA and MAQ results **(A)** CNV-NGS rappresentative images, the red box indicates the BRCA1 exon 20 deletion, exon 21-22 deletion and exon 24 deletion, sequencing reads of *BRCA1* (red) and *BRCA2* (blue) were normalized with sample ID (sid) tag sequencing reads (green). **(B)** MLPA results: the probes relative to exon 20, 21-22 and 24 show a RPR value about 0.5 compared to normal range value of reference probes (normal range : 0.7 – 1.3 red and blue line), blue rhombus represents the 95% confidence interval over the reference samples for each probe. The .collected data were analyzed using Coffalyser.NET Software (MRC Holland). **(C)** MAQ results show the 0.57 DQ value relative to BRCA1 exon 20, 0.53 and 0.51 for exon 21-22 and 0.53 for exon 24. The gray profiles are obtain on reference DNA sample, (normal range 0.75 – 1.3).

DNA was analyzed for point mutations and CNVs by a single workflow. PGM sequencing produced an average of 244,000 reads per patients, the mean read length being 110 bp. The average read depth per sample was 1220X, with a mean percentage of reads on target of 95%. The mean percentage of regions of interest (ROI) covered at least by 100X was 100%, and uniformity of base coverage by 99.5%. Details for each samples of the sequencing metrics are reported in Supplementary Table 1. Among 81 women with breast cancer who pursued genetic testing, 19 allelic variants were identified (detection rate 23%), 14 pathogenic (P) variants (74%, 14/19) including one splicing site mutation, one nonsense, one missense, 9 frameshifts, and three CNVs, all reported in the BRCA Exchange database (http://brcaexchange.org/); five mutations are classified as variants of uncertain significance (VUS) (Table 1). All samples analyzed for CNVs by the VCIB (Variability Correction Informatics Baseline) algorithm showed a confidence score > 20, indicating a high quality call of CNVs (Table 2). All results obtained by NGS-CNV detection were confirmed by MLPA (Supplementary Figure 1). Sixty-nine samples out 81 were negative for big indels (Supplementary Figure 2), while three samples (about 4%) showed an exon 20 deletion, exons 21 to 22 deletion and exon 24 deletion on the *BRCA1* gene respectively, with relative peak ratio (RPR) values about 0.5 (normal range 0.7-1.3). The identified CNVs were confirmed by the MAQ test, the dosage quotient (DQ) values were 0,57 for exon 20 deletion, 0.53-0.51 for exon 21-22 deletion and 0.53 for exon 24 deletion (normal range 0.75-1.3). Comparison between the results obtained through NGS-CNVs, MLPA and MAQ technology are summarized in Figure 1.

**Table 1:** Indel and SNV variants detected by Oncomine™ BRCA Panel

Gene	Transcript	Locus	Coding	Protein	Function	dbSNP	Clinical Significance	Enigma Classification	SampleID
*BRCA1*	NM_007294.3	chr17:41267797	c.81-1G>C	p.?	unknown	rs80358018	Pathogenic	C 5	P17
*BRCA1*	NM_007294.3	chr17:41243725	c.3823A>G	p.Ile1275Val	missense	rs80357280	Uncertain significance	C 3	P05
*BRCA1*	NM_007294.3	chr17:41245667	c.1881C>G	p.Val627Val	synonymous	rs80356838	Uncertain significance	C 3	P07
*BRCA1*	NM_007294.3	chr17:41244526	c.3018_3021delTTCA	p.His1006Glnfs*17	frameshiftDeletion	rs80357749	Pathogenic	C 5	P08
*BRCA1*	NM_007294.3	chr17:41243788	c.3756_3759delGTCT	p.Ser1253Argfs*10	frameshiftDeletion	rs80357868	Pathogenic	C 5	P11
*BRCA1*	NM_007294.3	chr17:41244262	c.3285delA	p.Lys1095Asnfs*14	frameshiftDeletion	rs397509051	Pathogenic	C 5	P16
*BRCA1*	NM_007294.3	chr17:41228628	c.4361T>C	p.Val1454Ala	missense	rs587782606	Uncertain significance	C 3	P13
*BRCA1*	NM_007294.3	chr17:41209079	c.5266dupC	p.Gln1756Profs*74	frameshiftInsertion	rs80357906	Pathogenic	C 5	P04
*BRCA1*	NM_007294.3	chr17:41215920	c.5123C>A	p.Ala1708Glu	missense	rs28897696	Pathogenic	C 5	P10
*BRCA2*	NM_000059.3	chr13:32905069	c.700delT	p.Ser234Profs*7	frameshiftDeletion	rs80359630	Pathogenic	C 5	P12
*BRCA2*	NM_000059.3	chr13:32907102	c.1487C>T	p.Ser496Phe	missense	rs397507269	Uncertain significance	C 3	P01
*BRCA2*	NM_000059.3	chr13:32906458	c.846_847delCA	p.Ile283Trpfs*11	frameshiftDeletion	rs886040776	Pathogenic	C 5	P15
*BRCA2*	NM_000059.3	chr17:32913381	c.4889C>G	p.Ser1630Ter	nonsense	rs80358711	Pathogenic	C 5	P18
*BRCA2*	NM_000059.3	chr13:32915053	c.6566dupA	p.Asn2189Lysfs*8	frameshiftInsertion	rs397507373	Pathogenic	C 5	P02
*BRCA2*	NM_000059.3	chr13:32914953	c.6468_6469delTC	p.Gln2157Ilefs*18	frameshiftDeletion	rs80359596	Pathogenic	C 5	P09
*BRCA2*	NM_000059.3	chr13:32944593	c.8386C>T	p.Pro2796Ser	missense	rs146120136	Uncertain significance	C 3	P14

Abbreviations: dbSNP: Single Nucleotide Polymorphisms database (www.ncbi.nlm.nih.gov/SNP); ENIGMA (Evidence-based Network for the Interpretation of Germline Mutant Alleles) http://www.enigmaconsortium.org: C5: Class 5 – (there is significant evidence to suggest that this variant is a dominant high-risk pathogenic variant, C3: Class 3 (there is insufficient evidence, molecular or otherwise, to be classified as a high-risk pathogenic variant, thus requires further investigation.

**Table 2:** Large rearrangements detected by NGS-CNV analysis in P03, P06 and P19 patients

Sample ID	Locus	Type	CNV Subtype	Call	Genes	CytoBand	Length	Variant Class	Copy Number	CNV Confidence
P03	chr13:32890490	CNV	REF	exon 2-27	*BRCA2*	13q13.1(32890490-32972932)x2	82.442kb		2	100
	**chr17:41197601**	**CNV**	**BigDel**	**exon 24**	***BRCA1***	**17q21.31(41197601-41197870)x1**	**269kb**	**exon deletion**	**1**	**30.53**
	chr17:41199538	CNV	REF	exon 2-23	*BRCA1*	17q21.31(41199538-41276123)x2	76.585kb		2	100
P06	chr13:32890490	CNV	REF	exon 2-27	*BRCA2*	13q13.1(32890490-32972932)x2	82.442kb		2	100
	chr17:41197601	CNV	REF	exon 21-24	*BRCA1*	17q21.31(41197601-41203234)x2	5.633kb		2	69.58
	**chr17:41208956**	**CNV**	**BigDel**	**exon 20**	***BRCA1***	**17q21.31(41208956-41209231)x1**	**275kb**	**exon deletion**	**1**	**78.56**
	chr17:41215248	CNV	REF	exon 2-19	*BRCA1*	17q21.31(41215248-41276123)x2	60.875kb		2	100
P19	chr13:32890490	CNV	REF	exon 2-27	*BRCA2*	13q13.1(32890490-32972932)x2	82.442kb		2	100
	chr17:41197601	CNV	REF	exon 23-24	*BRCA1*	17q21.31(41197601-41199764)x2	2.163kb		2	34.61
	**chr17:41201009**	**CNV**	**BigDel**	**exon 21-22**	***BRCA1***	**17q21.31(41201009-41203234)x1**	**2.225kb**	**exon deletion**	**1**	**100**
	chr17:41208956	CNV	REF	exon 2-20	*BRCA1*	17q21.31(41208956-41276123)x2	67.167kb		2	100

Abbreviations: REF: read count matches with the reference baseline; BigDel: deletion of at least one exon; CNV confidence: The confidence score is the probability that the number of copies of the region of interest is different from 2, which is the normal value. A high confidence score indicates a higher probability that the identified variant is a true positive. A confidence score of 10 or 20 or higher indicates a high quality copy number variant's call.

### Perfomance of the single workflow for SNVs, indels and CNVs detection

The single Oncomine workflow used for *BRCA1/2* genetic test allowed us to detect simultaneous all type of variants on *BRCA1/2* genes. Using this single integrated workflow on single platform, turnaround time of mutation testing was reduced. This approach allows to a single technician to perform the complete *BRCA1/2* analysis in a series of 16 patients (collected in two weeks) making the diagnostic reports available for the clinician in 25 working days following blood sampling, compared to 45 days required with separate workflows for point mutations and CNVs. To assess the sensitivity and specificity in the detection of SNVs and indels, we compared the variant calling results from four DNA samples, pre-tested by Ion AmpliSeq Panel to detect germline variants, with the results obtained by Oncomine® BRCA1/2 Panel. The comparison was restricted to the ROI regions (*BRCA1/2* coding regions +/- 20 bp) covered by both panels’ designs. All variants previously called by Ion AmpliSeq panel were successfully confirmed using the Oncomine® BRCA1/2 Panel, except for a false call in a homolymeric region of the BRCA2 gene (Supplementary Table 2).

Finally, to check the performance of NGS-CNVs detection, all samples analyzed with VCIB algorithm of Ion Reporter Server System 5.6, were re-analyzed by MLPA analysis. In addition, the CNVs identified in tree samples on *BRCA1* gene were analyzed by MAQ technique. CNV NGS data compared to MLPA and/or MAQ revealed a 100% of sensitivity, specificity and accuracy (Supplementary Table 3).

## DISCUSSION

The mutational spectrum of *BRCA1/2* is large, including different classes of variants, such as SNVs, indels and LGRs. Fast test results are crucial when patients with breast/ovarian cancer and their unaffected family members must be addressed to the appropriate therapy and surveillance respectively. Sanger sequencing and MLPA so far represented the gold standard for the complete *BRCA1/2* testing. However the possibility of analyzing all types of mutations through a single workflow offered by the NGS is appealing. Therefore several research groups have tested different NGS platforms for CNVs detection by implementing and validating customized workflow [21, 28–30].

In this study, we evaluated the possibility of using the PGM platform for the simultaneous analysis of indels, SNVs, and CNVs in *BRCA1/2* genes, by the Oncomine™ BRCA Research assay and Ion Reporter 5.6 Software, which includes two different algorithms for different classes of variant calling. The Oncomine™ BRCA Panel replaced the previously used Ion Ampliseq™ Panel on the PGM platform for *BRCA1/2* diagnostic testing. Another study showed a better performance of the Oncomine™ BRCA Research assay compared with the Ion Ampliseq™ BRCA Panel in terms of ability to identify deletions at homopolymer sites, on target mapped reads, reduction of low-quality mapped reads and false-negative results. In the same study, using the Oncomine Panel a large deletion was identified, subsequently confirmed by MLPA [31]. The NGS-CNV analysis requires a complete validation of the "baseline", which is crucial to obtain accurate and reliable results for clinical applications. For this purpose, we used the VCIB algorithm to build the baseline and set up basic parameters. To validate NGS accuracy on CNV detection we selected forty-eight samples, without big indels on *BRCA1/2* genes when tested by MLPA. Using a single workflow, we identified on *BRCA1/2* genes three large rearrangements and 16 point mutations. The clinical features of *BRCA1/2* carriers mutations are summarized in Supplementary Tables 4 and 5. Seventy-eight out of eighty-one samples were normal for *BRCA1/2* copy number variations (Figure 1, Supplementary Figure 2) Results obtained by the NGS-CNV algorithm were confirmed with MLPA and pathogenic CNVs were also confirmed with MAQ, with 100% concordance. For what concerns reduced costs, they have been mainly obtained by using a single protocol and reagents for the complete analysis of *BRCA1/2* genes. Moreover, the baseline implemented in the bioinformatics pipeline for the NGS-CNVs analysis does not need to insert additional control samples for each analysis, further reducing cost effective for a single sample; for the library preparations a very low input concentration of DNA is required compared with MLPA analysis. Using this workflow also the time of analysis has been drastically reduced obtaining two analyzes in a single solution and avoiding to introduce further errors and limiting contamination problems. We can conclude that NGS-CNV could be used as valid and safe alternative to MLPA assay; the NGS approach for CNV analysis could represent an effective procedure to apply to all patients in routine *BRCA1/2* molecular screening. Comprehensive and accurate analysis of *BRCA1/2* genes is essential for individual and family genetic counseling in hereditary breast and ovarian cancers [32], as well as for establishing drug therapy with poly(ADP-ribose) (PARP) polymerases inhibitors in patients with high-grade serous ovarian cancer [33, 34]. Therefore, reducing the timing of mutation screening becomes fundamental in clinical oncology. We confirm in this study the accuracy and precision of NGS workflow for simultaneous detection of CNVs and point mutations of *BRCA1/2* genes, suggesting the use of this technological advancement in the diagnostic-therapeutic and care assessment of hereditary breast and ovarian cancer.

## MATERIALS AND METHODS

### Patients

A consecutive series of 81 Italian women with breast/ovarian cancer and/or positive family history have been enrolled in this study between July and December 2017, after receiving genetic counseling at U.O.C Medical Genetics and Cellular Diagnostic of Department of Clinical and Molecular Medicine, “Sapienza” University of Rome. The eligibility of patients for *BRCA1/2* testing was evaluated using BRCAPRO 5.0 model (University of Texas http://www.stat.duke.edu/~gp/brcapro.html) and/or for the presence of specific anamnestic criteria based on Italian guidelines from the *Operational program 2016-2018, Lazio Region decree: DCA 52/201* that identify the high-risk woman for being *BRCA* mutation carriers. All tested individuals signed an informed consent for genetic research. Investigation has been conducted in accordance with the 1964 Helsinki Declaration.

### Mutational analysis

A single NGS platform, the Personal Genome Machine (PGM) (Thermo Fisher Scientific, Carlsbad, CA, USA), has been used for the simultaneous detection of point mutations and CNVs in *BRCA1/2* genes. For this purpose Oncomine™ BRCA Research Assay (Thermo Fisher Scientific), containing 265 primers pairs in two pools was performed according to the manufacturer's protocol on PGM machine. Genomic DNA was extracted from whole EDTA blood using a commercially available kit (Invitrogen, Pure link Genomic DNA by Thermo Fisher Scientific) and quantified using Qubit ds DNA HS Assay Kit on Qubit 3.0 Fluorimeter (Invitrogen). The detected variants are classified based on the criteria of the ENIGMA (Evidence-based Network for the Interpretation of Germline Mutant Alleles) consortium (https://enigmaconsortium.org) and described as recommended by Human Genome Variation Society (https://www.hgvs.org/) using as RefSeq: NM_007294.2 and NM_000059.3. Point mutations classified as P and VUS have been confirmed by Sanger sequencing, using the BigDye Terminator v3.1 sequencing kit and the ABI PRISM 3130 Genetic Analyzer (Life Technologies). To confirm the results obtained by NGS-CNV workflow, all the samples analyzed by Ion Reporter Software 5.6 were tested with Multiplex Ligation-dependent Probe Amplification (MLPA) and/or Multiplex Amplicon Quantification (MAQ).

*BRCA1/2* MLPA analysis was performed using P002-D1 and P045-C1 SALSA MLPA kits (MRC-Holland, Amsterdam, the Netherlands) in according to manufacturer's instructions. DNA samples were diluted to final 50ng/ul concentration, and four normal controls were included in each MLPA analysis. The fragments analysis was performed using 3130 Genetic Analyzer (Applied Biosystem) with size standard GeneScan TM 500 Liz. Variations in peaks areas were analyzed using Coffalyser.Net (MRC-Holland, Amsterdam, the Netherlands).

The MAQ (v1.0 kit, Multiplicon, Niel, Belgium) is a straightforward method for the detection and analysis of copy number variations (CNVs). It consists of the simultaneous PCR amplification of fluorescently labeled target amplicons of *BRCA1* and *BRCA2* exons followed by fragments analysis. The comparison of normalized peak area between test and reference sample results in a dosage quotient (DQ) indicating the copy of LGR. MAQ kit includes two Master reaction mix (Plex A and Plex B) containing primer for 55 *BRCA1/2* amplicons target (TA) and 17 control amplicons (CA). The fragment analysis was run on 3130 Genetic Analyzer (Applied Biosystem) and for analysis results was used the MAQ-S v2.0 software (Multiplicon, Niel Belgium).

### Library preparation

The Oncomine™ Panel used for library preparation, cover 100% of the coding sequences of *BRCA1* and *BRCA2*, including all splice sites with an average of 64 bp extensions from the intron junctions. In according to the manufacturer's instructions, 10ng of DNA isolated from whole blood per target amplification reaction (20ng total) were used to generate the sequencing libraries with two premixed pools of 265 primers. Briefly, after target amplification in 10ul reactions, pool 1 and pool 2 amplification reactions are combined into new wells in the plate. After partial digestion of primers, ligation of barcode adapters, and amplicon purification, barcoded libraries are quantified and diluted to 100pM concentration, and combined before template preparation. Clonal amplification of the libraries was carried out by emulsion PCR using Ion PGM™ Hi-Q™ View OT2 Kit on Ion OneTouch 2 Instrument and the Ion OneTouch ES (Enrichment System) (Thermo Fisher Scientific) to produce high-quality Ion Sphere™ particles for use in combination with the Ion PGM™ Hi-Q™ View Sequencing Kit. Finally, the prepared libraries were then sequenced on an Ion PGM™ System platform, using Ion 316™ Chip v2 BC. Sequencing data analysis was performed using Torrent Suite version 5.0.5 and Ion Reporter version 5.6 (Thermo Fisher Scientific).

### SNVs, indel and CNVs detections

The Torrent Suite Software running in Torrent Server (DEL T7500 OS Ubuntu 10.04 LTS) has been used to process raw data acquired by PGM. The generated raw sequence data, in FASTQ format, have been aligned to the hg19 human reference genome using the Torrent Mapping Alignment Program. The base calls, in SFF and FASTQ file formats were used for downstream analysis, containing per-base quality scores. Following the analysis, the annotation of single nucleotide variants, indels and CNVs was performed using the Ion Reporter Server System v.5.6. The analysis of the CNVs required the setting of the integrated software for germline CNVs analysis. In the proprietary algorithm used to call copy number changes in individual exons, named VCIB (Variability Correction Informatics Baseline), has been inserted a baseline consisting of samples normal for *BRCA1/2* CNVs. The selection of samples for the baseline was made between those previously analyzed for all type of *BRCA1/2* mutations, using Oncomine BRCA Panel for point mutations analysis on PGM and the MLPA for CNVs detection on Sanger sequencer. Among these, we selected 48 samples without big indels in the *BRCA1/2* genes, with a value of mapped reads > 100,000 and a MAPD (Median of the Absolute values of all Pairwise Differences) <0.5. The MAPD is a metric that evaluates whether panel data can be used for CNV analysis. The Copy Number estimates are made by VCIB algorithm counting reads for each amplicon, making adjustments to account for certain types of variability like those derived from imbalance pool. The Sequence data were evaluated using Integrative Genomics Viewer (IGV) (http://software.broadinstitute.org/software/igv/).

## SUPPLEMENTARY MATERIALS FIGURES AND TABLES





## Abbreviations

BigDel: deletion of at least one exon
CNV: copy number variation
FISH: fluorescent in situ hybridization
INDEL: single or multiple insertion/deletion
LGR: large genomic rearrangement
MAPD: median of the Absolute values of all Pairwise Differences
MAQ: multiplex amplicon quantification
MLPA: multiplex ligation probe amplification
NGS: next generation sequencing
P: pathogenic
PARP: poly(ADP-ribose) polymerases
PGM: personal genome machine
REF: read count matches with the reference baseline
ROI: regions of interest
RPR: relative peak ratio
SNV: single nucleotide variant
VCIB: variability correction informatics baseline
VUS: variant of uncertain significance
