# Supplementary material for: Rapid detection of copy number variations and point mutations in BRCA1/2 genes using a single workflow by ion semiconductor sequencing pipeline
Source: Oncotarget. 2018 Sep 14;9(72):33648–55. doi: 10.18632/oncotarget.26000 (PMC6154752; doi:10.18632/oncotarget.26000)
Supplement: Supplementary file 1 [file oncotarget-09-33648-s001.pdf]

Rapid detection of copy number variations and point mutations in *BRCA1/2* genes using a single workflow by ion semiconductor sequencing pipeline

SUPPLEMENTARY MATERIALS

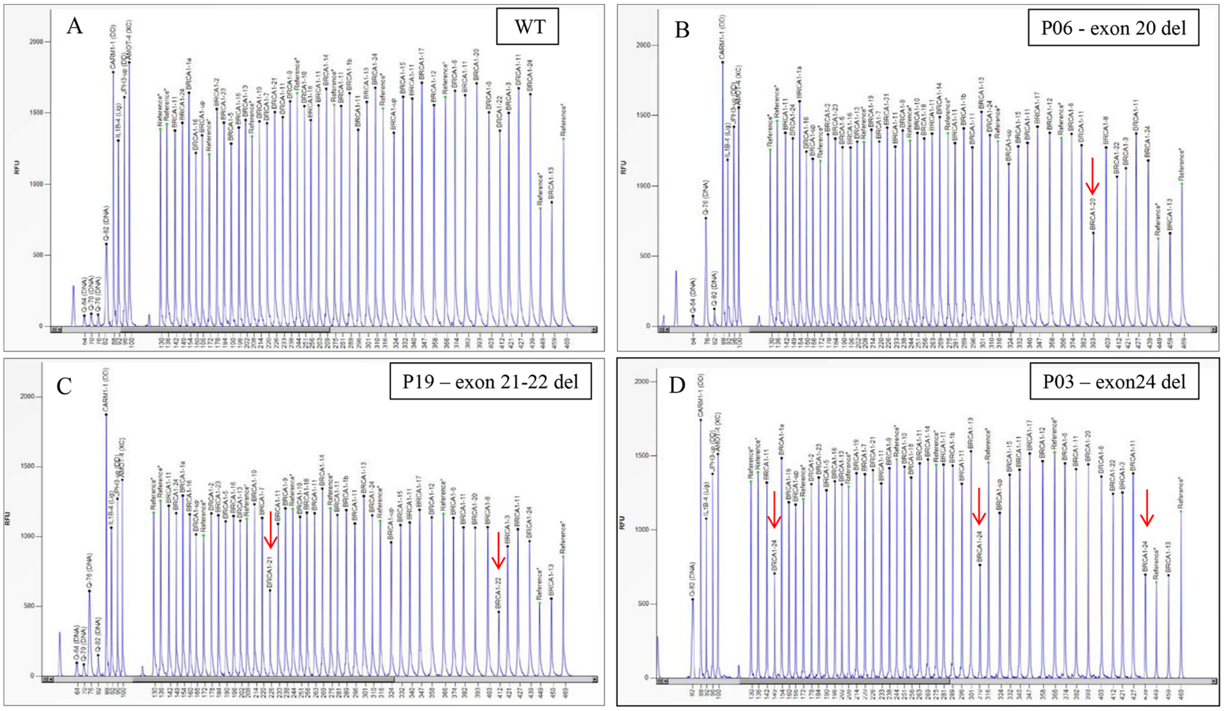

Supplementary Figure 1: *BRCA1* MLPA electropherogram showing aberrant profiles in P06 **(B),** P19 **(C),** and P03 **(D)** patients. Red arrow show respectively exon 20, 21-22 and 24 deletion of *BRCA1* gene. **(A)** wild-type sample (WT).

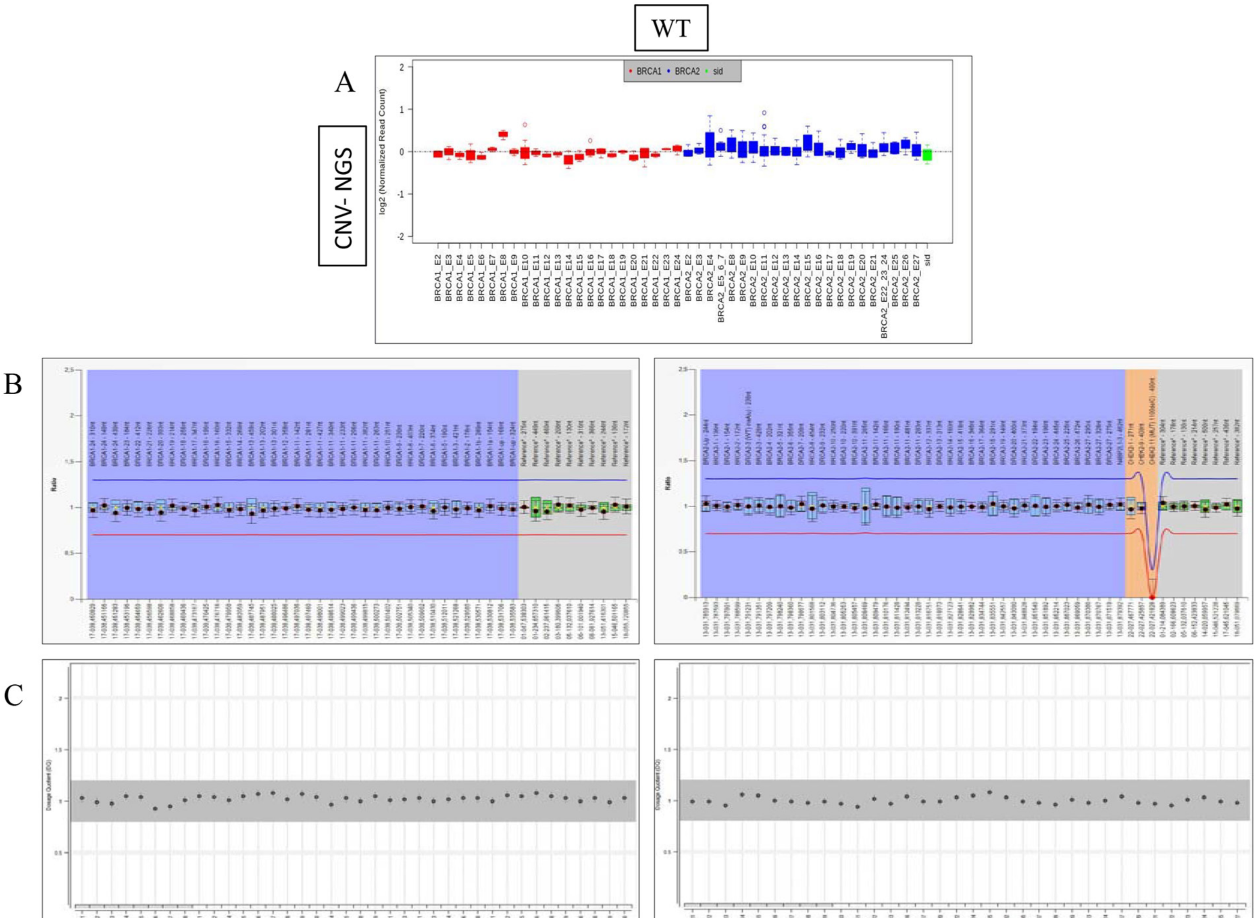

Supplementary Figure 2: Representative images from wild type sample for *BRCA1* and *BRCA2* CNVs **(A)** detected by Oncomine™ BRCA Research Assay compared with MLPA **(B)** and MAQ **(C)** results.

Supplementary Table 1: Sequencing metrics by NGS Analysis

Sample ID	Mapped Reads	Reads on Target (%)	ROI Mean Coverage	Uniformity (%)	ROI ≥ 100x (%)
P01	336454	96.54%	1510	100.00%	100%
P02	241196	96.31%	1078	100.00%	100%
P03	292299	96.60%	1302	100.00%	100%
P04	287979	96.29%	1290	100.00%	100%
P05	187414	96.22%	827.0	100.00%	100%
P06	172847	96.10%	763.1	100.00%	100%
P07	224832	96.64%	978.5	99.86%	100%
P08	258694	95.23%	1126	98.97%	100%
P09	311970	95.95%	1354	100.00%	100%
P10	229288	97.08%	1022	96.68%	100%
P11	190743	96.72%	757.8	93.64%	100%
P12	231776	95.94%	1047	100.00%	100%
P13	232925	96.04%	1051	100.00%	100%
P14	240933	96.47%	1098	99.71%	100%
P15	214566	96.20%	968.0	100.00%	100%
P16	297898	96.23%	1348	99.88%	100%
P17	247334	96.26%	1116	100.00%	100%
P18	248036	96.35%	1102	99.81%	100%
P19	277654	96.10%	1343.3	100%	100%
P20	324343	96.44%	1454	100.00%	100%
P21	293070	96.74%	1312	99.93%	100%
P22	263238	96.62%	1174	100.00%	100%
P23	277775	96.11%	1240	100.00%	100%
P24	193897	96.17%	857.4	100.00%	100%
P25	162927	96.41%	720.3	100.00%	100%
P26	171627	96.21%	763.2	100.00%	100%
P27	157030	96.42%	698.5	100.00%	100%
P28	206585	96.36%	910.3	99.94%	100%
P29	177664	96.45%	784.9	100.00%	100%
P30	188964	96.37%	837.2	100.00%	100%
P31	218852	96.16%	949.7	100.00%	100%
P32	246094	96.03%	1066	100.00%	100%
P33	259323	95.83%	1133	99.60%	100%
P34	312732	95.71%	1363	99.61%	100%
P35	340689	95.87%	1477	100.00%	100%
P36	248625	95.56%	1068	99.88%	100%
P37	213077	96.33%	877.5	99.64%	100%
P38	214036	96.46%	880.2	99.19%	100%
P39	226929	96.57%	945.8	99.86%	100%
P40	232595	96.32%	967.4	99.98%	100%
P41	221640	96.24%	921.5	99.94%	100%
P42	202101	96.14%	833.2	99.53%	100%
P43	225071	96.29%	934.6	99.95%	100%
P44	219332	97.15%	886.7	95.66%	100%
P45	243500	96.27%	976.4	93.11%	100%
P46	207845	96.92%	834.0	93.03%	100%
P47	216866	96.76%	881.1	96.29%	100%
P48	239820	95.93%	1084	100.00%	100%
P49	302854	96.06%	1369	100.00%	100%
P50	273885	95.87%	1235	100.00%	100%
P51	282737	95.88%	1272	100.00%	100%
P52	287967	96.05%	1298	100.00%	100%
P53	288181	96.18%	1303	100.00%	100%
P54	241094	96.09%	1097	99.72%	100%
P55	213296	96.28%	962.6	100.00%	100%
P56	221605	95.90%	995.3	99.72%	100%
P57	208714	96.42%	941.2	100.00%	100%
P58	222400	96.31%	1000	99.93%	100%
P59	187842	90.77%	789.2	100.00%	100%
P60	228352	96.07%	1026	100.00%	100%
P61	278907	96.44%	1263	99.88%	100%
P62	325182	96.25%	1453	99.93%	100%
P63	348784	96.28%	1580	100.00%	100%
P64	265044	96.24%	1195	100.00%	100%
P65	275661	96.52%	1247	99.95%	100%
P66	310945	96.41%	1409	99.88%	100%
P67	219285	96.69%	1004	99.60%	100%
P68	216653	96.49%	987.7	99.81%	100%
P69	166257	96.55%	755.1	99.88%	100%
P70	226062	96.30%	1024	99.98%	100%
P71	228168	96.50%	1036	99.88%	100%
P72	262871	96.84%	1206	99.88%	100%
P73	287340	96.55%	1309	100.00%	100%
P74	182150	96.52%	807.8	99.66%	100%
P75	192937	96.30%	859.1	100.00%	100%
P76	273590	95.67%	1220	85.28%	100%
P77	391564	96.32%	1724	99.20%	100%
P78	272467	96.01%	1202	99.92%	100%
P79	314131	96.17%	1387	99.92%	100%
P80	92445	95.72%	411.7	99.99%	100%
P81	77652	96.10%	343.3	100.00%	100%

The distribution of average coverage depth for all exon targets,displayed by sample. Regions of interest (ROI).

Supplementary Table 2: Variant Calling comparison between Ampliseq Panel and Oncomine™ BRCA Panel

See Supplementary File 1

Supplementary Table 3: Comparison of copy number analysis data: detected with NGS-CNV, MLPA, MAQ

Sample ID	NGS-CNV	MLPA	MAQ
P01	WT	WT	ND
P02	WT	WT	ND
P03	exon 24del	exon 24del	exon 24del
P04	WT	WT	ND
P05	WT	WT	ND
P06	exon 20del	exon 20del	exon 20del
P07	WT	WT	ND
P08	WT	WT	ND
P09	WT	WT	ND
P10	WT	WT	ND
P11	WT	WT	ND
P12	WT	WT	ND
P13	WT	WT	ND
P14	WT	WT	ND
P15	WT	WT	ND
P16	WT	WT	ND
P17	WT	WT	ND
P18	WT	WT	ND
P19	exon 21-22del	exon 21-22del	exon 21-22del
P20	WT	WT	ND
P21	WT	WT	ND
P22	WT	WT	ND
P23	WT	WT	ND
P24	WT	WT	ND
P25	WT	WT	ND
P26	WT	WT	ND
P27	WT	WT	ND
P28	WT	WT	ND
P29	WT	WT	ND
P30	WT	WT	ND
P31	WT	WT	ND
P32	WT	WT	ND
P33	WT	WT	ND
P34	WT	WT	ND
P35	WT	WT	ND
P36	WT	WT	ND
P37	WT	WT	ND
P38	WT	WT	ND
P39	WT	WT	ND
P40	WT	WT	ND
P41	WT	WT	ND
P42	WT	WT	ND
P43	WT	WT	ND
P44	WT	WT	ND
P45	WT	WT	ND
P46	WT	WT	ND
P47	WT	WT	ND
P48	WT	WT	ND
P49	WT	WT	ND
P50	WT	WT	ND
P51	WT	WT	ND
P52	WT	WT	ND
P53	WT	WT	ND
P54	WT	WT	ND
P55	WT	WT	ND
P56	WT	WT	ND
P57	WT	WT	ND
P58	WT	WT	ND
P59	WT	WT	ND
P60	WT	WT	ND
P61	WT	WT	ND
P62	WT	WT	ND
P63	WT	WT	ND
P64	WT	WT	ND
P65	WT	WT	ND
P66	WT	WT	ND
P67	WT	WT	ND
P68	WT	WT	ND
P69	WT	WT	ND
P70	WT	WT	ND
P71	WT	WT	ND
P72	WT	WT	ND
P73	WT	WT	ND
P74	WT	WT	ND
P75	WT	WT	ND
P76	WT	WT	ND
P77	WT	WT	WT
P78	WT	WT	ND
P79	WT	WT	WT
P80	WT	WT	ND
P81	WT	WT	ND

WT: wilde type; ND:Not detected.

Supplementary Table 4: Clinical features and personal cancer history of CNV *BRCA1* mutation carriers

Sample ID	P06	P19	P03	
Age at diagnosis	54 y	35 y	38 y	43 y
**Ovarian cancer**				
Histologicsubtype	HGS			
Cancer stage	IC (T1c, N0, M0)			
Platinum sensitivity	yes			
Chemotherapy	Adjuvant			
**Breast cancer**				
Laterality		Right	Left	Right
Histology		MCB	IDC	IDC
Grade		G3	G3;	G2
ER-status		5%	0	0
PR status		5%	0	0
HER-2 Status		3+	3+	0
Triple negative breast cancer				Yes
T- Stage		ypT1b	pT1c	pT1c
N-stage		ypN0	pN0	pN0
Overall TNM Stage		II	IA	IA
Adjuvantradiation			Yes	Yes
Chemotherapy		neoadjuvant	Adjuvant	Adjuvant
Type of surgery		mastectomy	Lumpectomy	Lumpectomy
***BRCA1/2* mutation status**	*BRCA1* ex 20del	*BRCA1* ex 21-22del	*BRCA1* ex 24del	
**Family History**	Not information	Yes	Yes	

Abbreviations: HGS OC: High Serous Ovarian Cancer; IDC: Invasive Ductal Carcinoma; MCB: Metaplastic Breast Cancer.

Supplementary Table 5: Clinical data of patients carrying *BRCA1/2* P and VUS variants

ID Sample	Age at diagnosis/observation	Family History	Tumor	Triple negative	
P01	48	Y	BC	N	
P02	48	Y	BC	N	
**P03**	38 - 43	Y	BBC	Y	Y
P04	43	Y	BC	N	
P05	35	Y	unaffected	N	
**P06**	54	no info	HGS OC	N	
P07	56	N	BC	Y	
P08	49 - 56	Y	BBC – HGS OC	Y	Y
P09	36	Y	unaffected	N	
P10	38	Y	BC	N	
P11	35	Y	BC	N	
P12	40 – 44	Y	BBC	N	N
P13	43	Y	BC	N	
P14	48	Y	BC	N	
P15	36	Y	BC	N	
P16	49	Y	OC	N	
P17	52- 53-60	Y	RMS - OC - BC	Y	
P18	53	Y	BC	N	
**P19**	35	Y	BC	N	

Abbreviations: BC: Breast Cancer, BBC: Bilateral breast cancer, OC: Ovarian cancer, RMS: Rhabdomyosarcoma P: pathogenic VUS: variant of uncertain significance.
