# Supplementary material for: Rapid detection of copy number variations and point mutations in BRCA1/2 genes using a single workflow by ion semiconductor sequencing pipeline
Source: Oncotarget. 2018 Sep 14;9(72):33648–55. doi: 10.18632/oncotarget.26000 (PMC6154752; doi:10.18632/oncotarget.26000)
Supplement: Supplementary file 2 [file oncotarget-09-33648-s002.docx]

**Supplementary Table 2**: Variant Calling comparison between Ampliseq Panel and Oncomine™ BRCA Panel

| **Sample ID: Crt01** | | **Ampliseq** | | **Oncomine** | |  | | | | | | |
| --- | --- | --- | --- | --- | --- | --- | --- | --- | --- | --- | --- | --- |
| **Gene** | **Transcript** | **# Locus** | **Type** | **# Locus** | **Type** | **Function** | **Protein** | **Coding** | **dbSNP** | **Clinical Significance** | **Enigma Classification** |  |
| *BRCA2* | NM_000059.3 | chr13:32890572 | SNV | chr13:32890572 | SNV | UTR variant | UTR variant | c.-26G>T | rs1799943 | Benign | C1 |  |
| *BRCA2* | NM_000059.3 | chr13:32911375 | SNV | chr13:32911375 | SNV | synonymous | p.Gln961Gln | c.2883G>A | rs11571655 | Benign | C1 |  |
| *BRCA2* | NM_000059.3 | chr13:32911888 | SNV | chr13:32911888 | SNV | synonymous | p.Lys1132Lys | c.3396A>G | rs1801406 | Benign | C1 |  |
| *BRCA2* | NM_000059.3 | chr13:32913055 | SNV | chr13:32913055 | SNV | synonymous | p.Leu1521Leu | c.4563A>G | rs206075 | Benign | C1 |  |
| *BRCA2* | NM_000059.3 | chr13:32915005 | SNV | chr13:32915005 | SNV | synonymous | p.Val2171Val | c.6513G>C | rs206076 | Benign | C1 |  |
| *BRCA2* | NM_000059.3 | chr13:32929232 | SNV | chr13:32929232 | SNV | synonymous | p.Ser2414Ser | c.7242A>G | rs1799955 | Benign | C1 |  |
| *BRCA2* | NM_000059.3 | chr13:32929387 | SNV | chr13:32929387 | SNV | missense | p.Val2466Ala | c.7397T>C | rs169547 | Benign | C1 |  |
| *BRCA2* | NM_000059.3 | chr13:32936646 | SNV | chr13:32936646 | SNV | intron variant | intron variant | c.7806-14T>C | rs9534262 | Benign | C1 |  |
| *BRCA1* | NM_007300.3 | chr17:41222948 | INDEL | chr17:41222948 | INDEL | frameshiftDeletion | p.Ser1676fs | c.5027_5045delCTGGCCTGACCCCAGAAGA | rs80359876 | Pathogenic | C5 |  |
| *BRCA1* | NM_007300.3 | chr17:41223094 | SNV | chr17:41223094 | SNV | missense | p.Ser1634Gly | c.4900A>G | rs1799966 | Benign | C1 |  |
| *BRCA1* | NM_007300.3 | chr17:41234470 | SNV | chr17:41234470 | SNV | synonymous | p.Ser1436 | c.4308T>C | rs1060915 | Benign | C1 |  |
| *BRCA1* | NM_007300.3 | chr17:41244000 | SNV | chr17:41244000 | SNV | missense | p.Lys1183Arg | c.3548A>G | rs16942 | Benign | C1 |  |
| *BRCA1* | NM_007300.3 | chr17:41244435 | SNV | chr17:41244435 | SNV | missense | p.Glu1038Gly | c.3113A>G | rs16941 | Benign | C1 |  |
| *BRCA1* | NM_007300.3 | chr17:41244936 | SNV | chr17:41244936 | SNV | missense | p.Pro871Leu | c.2612C>T | rs799917 | Benign | C1 |  |
| *BRCA1* | NM_007300.3 | chr17:41245237 | SNV | chr17:41245237 | SNV | synonymous | p.Leu771Leu | c.2311T>C | rs16940 | Benign | C1 |  |
| *BRCA1* | NM_007300.3 | chr17:41245466 | SNV | chr17:41245466 | SNV | synonymous | p.Ser694Ser | c.2082C>T | rs1799949 | Benign | C1 |  |
|  | | | | | | | | | | | | |
| **Sample ID: Crt02** | | **Ampliseq** | | **Oncomine** | |  | | | | | | |
| **Gene** | **Transcript** | **# Locus** | **Type** | **# Locus** | **Type** | **Function** | **Protein** | **Coding** | **dbSNP** | **Clinical Significance** | **Enigma Classification** |  |
| *BRCA2* | NM_000059.3 | chr13:32890572 | SNV | chr13:32890572 | SNV | UTR variant | c.-26G>T |  | rs1799943 | Benign | C1 |  |
| *BRCA2* | NM_000059.3 | chr13:32906547 | FP | / |  |  |  |  |  |  |  |  |
| *BRCA2* | NM_000059.3 | chr13:32911888 | SNV | chr13:32911888 | SNV | synonymous | p.Lys1132Lys | c.3396A>G | rs1801406 | Benign | C1 |  |
| *BRCA2* | NM_000059.3 | chr13:32913055 | SNV | chr13:32913055 | SNV | synonymous | p.Leu1521Leu | c.4563A>G | rs206075 | Benign | C1 |  |
| *BRCA2* | NM_000059.3 | chr13:32914529 | SNV | chr13:32914529 | SNV | nonsense | p.Lys2013Ter | c.6037A>T | rs80358840 | Pathogenic | C5 |  |
| *BRCA2* | NM_000059.3 | chr13:32915005 | SNV | chr13:32915005 | SNV | synonymous | p.Val2171Val | c.6513G>C | rs206076 | Benign | C1 |  |
| *BRCA2* | NM_000059.3 | chr13:32929232 | SNV | chr13:32929232 | SNV | synonymous | p.Ser2414Ser | c.7242A>G | rs1799955 | Benign | C1 |  |
| *BRCA2* | NM_000059.3 | chr13:32929387 | SNV | chr13:32929387 | SNV | missense | p.Val2466Ala | c.7397T>C | rs169547 | Benign | C1 |  |
| *BRCA2* | NM_000059.3 | chr13:32936646 | SNV | chr13:32936646 | SNV | intron variant |  | c.7806-14T>C | rs9534262 | Benign | C1 |  |
|  | | | | | | | | | | | |  |
| **Sample ID: Crt03** | | **Ampliseq** | | **Oncomine** | |  | | | | | | |
| **Gene** | **Transcript** | **# Locus** | **Type** | **# Locus** | **Type** | **Function** | **Protein** | **Coding** | **dbSNP** | **Clinical Significance** | **Enigma Classification** |  |
| *BRCA2* | NM_000059.3 | chr13:32890572 | SNV | chr13:32890572 | SNV | UTR variant | c.-26G>T |  | rs1799943 | Benign | C1 |  |
| *BRCA2* | NM_000059.3 | chr13:32903604 | INDEL | chr13:32903604 | INDEL | frameshiftDeletion | p.Val220fs | c.658_659delGT | rs80359604 | Pathogenic | C5 |  |
| *BRCA2* | NM_000059.3 | chr13:32911888 | SNV | chr13:32911888 | SNV | synonymous | p.Lys1132Lys | c.3396A>G | rs1801406 | Benign | C1 |  |
| *BRCA2* | NM_000059.3 | chr13:32912299 | SNV | chr13:32912299 | SNV | synonymous | p.Val1269Val | c.3807T>C | rs543304 | Benign | C1 |  |
| *BRCA2* | NM_000059.3 | chr13:32913055 | SNV | chr13:32913055 | SNV | synonymous | p.Leu1521Leu | c.4563A>G | rs206075 | Benign | C1 |  |
| *BRCA2* | NM_000059.3 | chr13:32915005 | SNV | chr13:32915005 | SNV | synonymous | p.Val2171Val | c.6513G>C | rs206076 | Benign | C1 |  |
| *BRCA2* | NM_000059.3 | chr13:32929232 | SNV | chr13:32929232 | SNV | synonymous | p.Ser2414Ser | c.7242A>G | rs1799955 | Benign | C1 |  |
| *BRCA2* | NM_000059.3 | chr13:32929387 | SNV | chr13:32929387 | SNV | missense | p.Val2466Ala | c.7397T>C | rs169547 | Benign | C1 |  |
| *BRCA2* | NM_000059.3 | chr13:32936646 | SNV | chr13:32936646 | SNV |  |  | c.7806-14T>C | rs9534262 | Benign | C1 |  |
| *BRCA1* | NM_007300.3 | chr17:41223094 | SNV | chr17:41223094 | SNV | missense | p.Ser1634Gly | c.4900A>G | rs1799966 | Benign | C1 |  |
| *BRCA1* | NM_007300.3 | chr17:41234470 | SNV | chr17:41234470 | SNV | synonymous | p.Ser1436Ser | c.4308T>C | rs1060915 | Benign | C1 |  |
| *BRCA1* | NM_007300.3 | chr17:41244000 | SNV | chr17:41244000 | SNV | missense | p.Lys1183Arg | c.3548A>G | rs16942 | Benign | C1 |  |
| *BRCA1* | NM_007300.3 | chr17:41244435 | SNV | chr17:41244435 | SNV | missense | p.Glu1038Gly | c.3113A>G | rs16941 | Benign | C1 |  |
| *BRCA1* | NM_007300.3 | chr17:41244936 | SNV | chr17:41244936 | SNV | missense | p.Pro871Leu | c.2612C>T | rs799917 | Benign | C1 |  |
| *BRCA1* | NM_007300.3 | chr17:41245237 | SNV | chr17:41245237 | SNV | synonymous | p.Leu771Leu | c.2311T>C | rs16940 | Benign | C1 |  |
| *BRCA1* | NM_007300.3 | chr17:41245466 | SNV | chr17:41245466 | SNV | synonymous | p.Ser694Ser | c.2082C>T | rs1799949 | Benign | C1 |  |
| *BRCA1* | NM_007300.3 | chr17:41246481 | SNV | chr17:41246481 | SNV | missense | p.Gln356Arg | c.1067A>G | rs1799950 | Benign | C1 |  |
|  | | | | | | | | | | | |  |
| **Sample ID: Crt04** | | **Ampliseq** | | **Oncomine** | |  |  |  |  |  |  |  |
| **Gene** | **Transcript** | **# Locus** | **Type** | **# Locus** | **Type** | **Function** | **Protein** | **Coding** | **dbSNP** | **Clinical Significance** | **Enigma Classification** |  |
| *BRCA2* | NM_000059.3 | chr13:32906729 | SNV | chr13:32906729 | SNV | missense | p.Asn372His | c.1114A>C | rs144848 | Benign | C1 |  |
| *BRCA2* | NM_000059.3 | chr13:32912299 | SNV | chr13:32912299 | SNV | synonymous | p.Val1269Val | c.3807T>C | rs543304 | Benign | C1 |  |
| *BRCA2* | NM_000059.3 | chr13:32913055 | SNV | chr13:32913055 | SNV | synonymous | p.Leu1521Leu | c.4563A>G | rs206075 | Benign | C1 |  |
| *BRCA2* | NM_000059.3 | chr13:32915005 | SNV | chr13:32915005 | SNV | synonymous | p.Val2171Val | c.6513G>C | rs206076 | Benign | C1 |  |
| *BRCA2* | NM_000059.3 | chr13:32929387 | SNV | chr13:32929387 | SNV | missense | p.Val2466Ala | c.7397T>C | rs169547 | Benign | C1 |  |
| *BRCA2* | NM_000059.3 | chr13:32936646 | SNV | chr13:32936646 | SNV |  |  | c.7806-14T>C | rs9534262 | Benign | C1 |  |
| *BRCA1* | NM_007300.3 | chr17:41223094 | SNV | chr17:41223094 | SNV | missense | p.Ser1634Gly | c.4900A>G | rs1799966 | Benign | C1 |  |
| *BRCA1* | NM_007300.3 | chr17:41234470 | SNV | chr17:41234470 | SNV | synonymous | p.Ser1436Ser | c.4308T>C | rs1060915 | Benign | C1 |  |
| *BRCA1* | NM_007300.3 | chr17:41244000 | SNV | chr17:41244000 | SNV | missense | p.Lys1183Arg | c.3548A>G | rs16942 | Benign | C1 |  |
| *BRCA1* | NM_007300.3 | chr17:41244435 | SNV | chr17:41244435 | SNV | missense | p.Glu1038Gly | c.3113A>G | rs16941 | Benign | C1 |  |
| *BRCA1* | NM_007300.3 | chr17:41244936 | SNV | chr17:41244936 | SNV | missense | p.Pro871Leu | c.2612C>T | rs799917 | Benign | C1 |  |
| *BRCA1* | NM_007300.3 | chr17:41245237 | SNV | chr17:41245237 | SNV | synonymous | p.Leu771Leu | c.2311T>C | rs16940 | Benign | C1 |  |
| *BRCA1* | NM_007300.3 | chr17:41245466 | SNV | chr17:41245466 | SNV | synonymous | p.Ser694Ser | c.2082C>T | rs1799949 | Benign | C1 |  |
| *BRCA1* | NM_007300.3 | chr17:41276044 | INDEL | chr17:41276047 | INDEL | frameshiftInsertion | p.Glu23fs | c.66_67insA | rs80357783 | Pathogenic | C5 |  |

CTR: control sample; FP: False positive; dbSNP: Single Nucleotide Polymorphisms database (www.ncbi.nlm.nih.gov/SNP); ENIGMA (Evidence-based Network for the Interpretation of Germline Mutant Alleles) http://www.enigmaconsortium.org: C5: Class 5 – (there is significant evidence to suggest that this variant is a dominant high-risk pathogenic variant, C3: Class 3 (there is insufficient evidence, molecular or otherwise, to be classified as a high-risk pathogenic variant, thus requires further investigation
